# Myd88 deficiency influences murine tracheal epithelial metaplasia and submucosal gland abundance

**DOI:** 10.1002/path.2876

**Published:** 2011-05-10

**Authors:** Adam Giangreco, Liwen Lu, Dawn J Mazzatti, Bradley Spencer-Dene, Emma Nye, Vitor Hugo Teixeira, Sam M Janes

**Affiliations:** 1 Centre for Respiratory Research, University College London, Rayne Institute5 University Street, London WC1E 6JF, UK; 2 Epithelial Cell Biology Laboratory, Cancer Research UK Cambridge Research InstituteRobinson Way, Cambridge CB2 0RE, UK; 3 Unilever DiscoverColworth Science Park, Sharnbrook MK44 1LQ, UK; 4 Experimental Histopathology Laboratory, Cancer Research UK London Research Institute44 Lincoln's Inn Fields, London WC2A 3PX, UK

**Keywords:** Myd88, mucus, trachea, secretion, injury, homeostasis

## Abstract

Tracheal epithelial remodelling, excess mucus production, and submucosal gland hyperplasia are features of numerous lung diseases, yet their origins remain poorly understood. Previous studies have suggested that NF-κB signalling may regulate airway epithelial homeostasis. The purpose of this study was to determine whether deletion of the NF-κB signalling pathway protein myeloid differentiation factor 88 (Myd88) influenced tracheal epithelial cell phenotype. We compared wild-type and Myd88-deficient or pharmacologically inhibited adult mouse tracheas and determined that in vivo Myd88 deletion resulted in increased submucosal gland number, secretory cell metaplasia, and excess mucus cell abundance. We also found that Myd88 was required for normal resolution after acute tracheal epithelial injury. Microarray analysis revealed that uninjured Myd88-deficient tracheas contained 103 transcripts that were differentially expressed relative to wild-type and all injured whole tracheal samples. These clustered into several ontologies and networks that are known to functionally influence epithelial cell phenotype. Comparing these transcripts to those expressed in airway progenitor cells revealed only five common genes, suggesting that Myd88 influences tracheal epithelial homeostasis through an extrinsic mechanism. Overall, this study represents the first identification of Myd88 as a regulator of adult tracheal epithelial cell phenotype. Copyright © 2011 Pathological Society of Great Britain and Ireland. Published by John Wiley & Sons, Ltd.

## Introduction

Airway mucus production is central to lung immunity and pathogen clearance. In humans, mucus is produced within specialized proximal airway secretory cells and appendages termed submucosal glands (SMGs) [1]. In contrast, mucus-producing cells and submucosal glands are rare in murine airways and are generally restricted to the most proximal regions of the tracheal epithelium [2, 3]. Previous studies indicate that murine mucus-producing cells develop via transdifferentiation (metaplasia) of tracheal Clara cell secretory protein (CCSP)-expressing (CE) cells or tracheal basal cells [4]. Balancing mucus production in human airways is crucial for airway patency and host defence. Mucus cell metaplasia, submucosal gland remodelling, and mucus plugging are associated with many chronic lung diseases including cystic fibrosis, chronic obstructive pulmonary disease, and severe asthma. It is therefore important to improve our understanding of mechanisms regulating submucosal gland formation, secretory cell differentiation, and mucus cell metaplasia.

Several groups have investigated mechanisms of submucosal gland development and mucus cell differentiation using murine transgenic and knockout models. These studies have determined that deletion of the transcription factor Lef1 inhibits tracheal submucosal gland formation [5] and that normal Lef1-dependent SMG induction is regulated by exogenous Wnt3a [6]. Murine models have also determined that ectodysplasin A receptor (Edar) activity is necessary for normal mucus cell differentiation and SMG development [7, 8]. Human *EDAR* mutations are known to inhibit airway mucus cell development and are associated with increased respiratory infection [9, 10]. Separately, two genetic loci on murine chromosomes 9 and 10 have been independently associated with tracheal SMG abundance and distribution [3]. These studies suggest that multiple genes and signalling networks may regulate airway submucosal gland differentiation and mucus cell metaplasia.

In addition to studies in murine models, it has been observed that human respiratory diseases with mucus cell metaplasia often exhibit NF-κB signalling pathway activation [11, 12]. NF-κB regulates numerous cellular and systemic processes including proliferation, differentiation, apoptosis, and inflammation [13]. The NF-κB pathway comprises five distinct transcription factor subunits (RelA, RelB, c-Rel, p50, and p52) that are normally inactive within the cytoplasm but which undergo nuclear translocation following IkB degradation. NF-κB signals are regulated by multiple ligands/receptor pairs including Toll-like receptors 1–9 (Tlr1–9), interleukin 1 receptor, tumour necrosis factor receptor (Tnfr), and Edar. Receptor signalling occurs either via myeloid differentiation factor 88 (Myd88)-dependent or via Myd88-independent pathways [13].

Genetically modified mouse models designed to elucidate how NF-κB signalling regulates airway mucus cell metaplasia have produced conflicting results. Epithelial-specific IκB super-repressor (IκB-SR) (NF-κB inhibited) transgenic mice display increased mucus metaplasia following chrysotile asbestos fibre exposure [14] but reduced metaplasia after ovalbumin sensitization and challenge [15]. Additional studies that involved *Myd88* deletion within bone marrow-derived cells (BMDCs) also exhibited increased airway epithelial hyperreactivity after challenge [16–19]. Conversely, transgenic mice with increased epithelial NF-κB activity display apparently normal mucus cell abundance [20].

In this study, we determined that *Myd88* deletion increased adult tracheal SMG abundance as well as mucus cell metaplasia and that Myd88 was required for normal resolution after acute tracheal epithelial injury. We also describe a unique tracheal gene expression profile associated with Myd88 deficiency that suggests potential regulatory mechanisms for mucus cell and submucosal gland metaplasia.

## Materials and methods

### Animal husbandry and tracheal injury

Adult, 4- to 6-month-old male and female C57/Bl6 wild-type and *Myd88* knockout (KO) mice were housed in individually ventilated cages on a 12 h light/dark cycle and allowed access to food and water *ad libitum*. Mice were kept in specific pathogen-free conditions and no infections were detected via sentinel screening during these studies. KO mice were generated as previously described [21], followed by six generations back-crossing to a wild-type C57/Bl6JOlaHsd congenic mouse strain (originally purchased from Harlan Laboratories, Huntington, UK). Knockout mice were maintained by KO × KO crossings; wild-type C57/Bl6JOlaHsd mice were maintained as a separate in-house colony. Adult mice were derived from at least three separate yet age-matched litters for all experiments. For pharmacological inhibition studies, IMG-2005 or a control peptide was prepared under aseptic conditions according to the manufacturer's protocol (Imgenex, San Diego, CA, USA). Mice were treated daily via intraperitoneal injection of 25 µg of IMG-2005 or control peptide for a total of 10 days. One hour prior to sacrifice, all mice received 10 mg BrdU/kg body weight.

In order to study tracheal repair, mice were anaesthetized with isofluorane and tracheas were damaged by oropharyngeal instillation of 10 µl of 2% polidocanol (Sigma, Dorset, UK). This established protocol results in rapid desquamation of tracheal epithelial cells within 24 h, followed by robust proliferation 3 days after injury, keratin 14 hyperplasia and transdifferentiation within 7–10 days [22], and complete repair within 30 days. A minimum *n* = 3 animals were used for each recovery time point. Mice were sacrificed by sodium pentobarbital overdose and all experiments were performed with approval of the UK Home Office (licence number 70/6077).

### Tissue preparation and immunostaining

Tissues were fixed overnight in 4% paraformaldehyde, processed, and sectioned longitudinally. Haematoxylin and eosin (H&E), periodic acid Schiff (PAS), Alcian blue, and Gram (bacterial) histochemical staining was performed using standard protocols on an automated staining system (TissueTek, Osaka, Japan).

Immunohistochemical and immunofluorescent staining of sections and air–liquid interface cell wholemounts followed standard conditions [23]. Primary antibodies included keratin 14 (rabbit; Thermo Scientific, Huntsville, AL, USA), CCSP (goat; provided by Dr Barry Stripp), BrdU (sheep; Abcam, Cambridge, USA), acetylated tubulin (mouse; Sigma), mucin 5AC (mouse; Sigma), 7/4 (neutrophils, rat; Abcam), and F4/80 (macrophages, rat; Abcam). Species-appropriate secondary antibodies included biotin-conjugated anti-rat and anti-sheep antibodies, streptavidin-HRP, and directly conjugated Alexafluor dyes (all Invitrogen, Carlsbad, CA, USA). For detection of apoptotic cells in paraffin sections, a DeadEnd colorimetric TUNEL system (Promega, Madison, WI, USA) was used according to the manufacturer's protocol. Images were obtained using a Leica TCS Tandem confocal at 10× or 20× objective magnification, as well as a Leica TCS-SPE confocal at 40× objective magnification (Leica Microsystems, Milton Keynes, UK).

### In vitro air–liquid interface (ALI) culture

Tracheal epithelial cells from five knockout and six wild-type mice were harvested using aseptic techniques, pooled, cleaned, digested in pronase, and cultured as previously described [24]. Enzyme isolated cells were counted and seeded in 12-well Transwell dishes (Corning Life Sciences, Amsterdam, The Netherlands) at 2 × 10^5^ cells per well in MTEC/plus [24]. Upon visually reaching confluence (day 8 post-plating), media were changed to MTEC/basic plus retinoic acid and cells were subsequently cultured at an air interface. All cultures contained visibly beating ciliated cells by day 19 and were fixed at this time.

### RNA preparation

Total RNA was prepared from whole tracheal homoge-nates suspended in 4 m guanidine isothiocyanate as previously described [25]. We prepared five biological replicates of each of four adult trachea sample groups: wild-type and knockout controls and 3-day-recovered, polidocanol-injured wild-type and knockout mice. All RNA samples were assessed for total RNA quality using an Agilent RNA 6000 NanoChip kit (Agilent Technologies, Santa Clara, CA, USA). Four of these wild-type and four knockout control samples were used for quantitative RT-PCR studies and normalized against 18S, β-actin, and GAPDH (see Supporting information, Supplementary methods).

### Gene expression microarrays

Labelled input RNA (2 µg) was prepared using an Agilent RNA amplification system according to the manufacturer's instructions [26]. We analysed RNA samples using an Agilent Hybridization Kit in conjunction with Agilent Mouse Oligo Arrays (Agilent Technologies). Hybridization was performed as previously described [26] and slides scanned with an Agilent G2565BA Microarray Scanner. Agilent G2567AA Feature Extraction and GenespringX 7.3 software was used for data extraction, quality control, and analysis. Transcripts that were classified as present in less than 50% of samples were excluded from analysis. We compared gene expression levels across all wild-type and KO samples and considered transcripts to be differentially expressed if they achieved a significance score of *p* < 0.01 by one-way, parametric ANOVA, followed by Benjamini and Hochberg multiple test correction. All microarray data are MIAME compliant (GSE17268).

### Comparative microarray analysis

We compared the expression profiles of genes that we identified as differentially expressed in Myd88-deficient, uninjured tracheal samples with a publicly available tracheal epithelial stem and progenitor cell profiles (GSE 15 724) [27]. Gene expression levels were plotted using a semi-logarithmic plot, with Myd88KO gene expression relative to wild-type samples plotted along the *X*-axis and the average stem or progenitor cell expression (K5/lectin double or single positive) relative to lectin plus keratin 5 negative (‘double negative’) cells along the *Y*-axis. We considered those genes exhibiting at least a two-fold increase in either the stem or the progenitor cells relative to double negatives as being significantly enriched. All genes identified in our comparison exhibited a *p* value less than or equal to 0.05 in the previously published study [27].

### Tissue morphometry and statistical analysis

Epithelial cell densities were determined as previously described by measuring the incidence of specific cell types as a function of basement membrane length [25, 28]. A minimum sample size of *n* = 3 was used for all morphometric sample analyses. Statistical significance of epithelial cell differentiation and abundance was determined using an unpaired Student's *t*-test for pairwise comparisons or ANOVA followed by Tukey's post-test pairwise comparison for multivariate sample comparisons.

## Results

### Myd88 deficiency promotes increased tracheal submucosal gland abundance and secretory cell metaplasia

**Figure 1:**
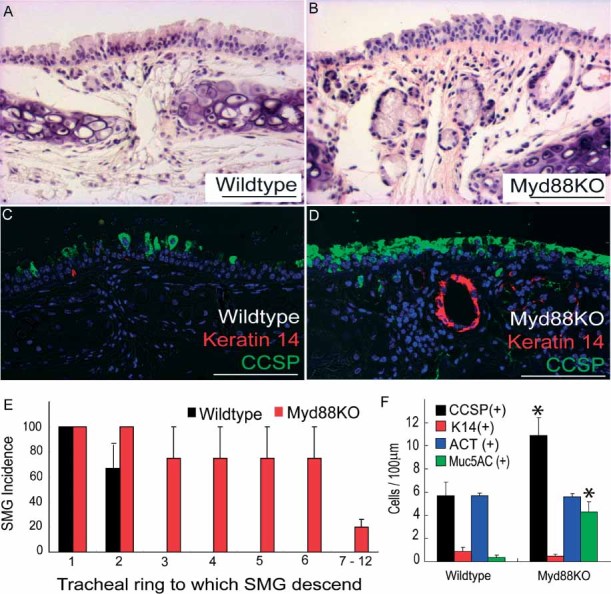
Myd88-deficient tracheas contain increased submucosal gland (SMG) abundance and secretory cell phenotypes. (A–D) Wild-type (A, C) and KO (B, D) tracheas were stained with haematoxylin and eosin (H&E; A, B) or keratin 14 (red) plus CCSP (green; C, D) to assess SMG abundance and epithelial cell phenotype. (E) Tracheal cartilaginous rings and SMGs were identified morphologically using H&E staining and SMGs classified according to the tracheal cartilaginous ring to which they descend. (F) Quantification of wild-type and Myd88KO tracheal epithelial CCSP (black bars), K14 (red bars), acetylated tubulin (ACT; blue bars), and Muc5AC (green bars) expressing cell abundance. Error bars (E, F) represent the standard error of the mean; asterisks denote significance at *p* < 0.05 (*n* = 5 mice per genotype). Scale bars are 100 µm (A–D) and DAPI was used as a nuclear counterstain (C, D)

In order to determine whether *Myd88* influenced epithelial cell phenotypes, we compared adult (4- to 6-month-old) C57/Bl6 wild type and Myd88 knockout (Myd88KO) tracheas. We used haematoxylin and eosin (H&E) staining to identify any histological differences between mice (Figures 1A and 1B), and immunofluorescent staining with the basal cell and submucosal gland (SMG) marker keratin 14 (K14), the epithelial cell marker Clara cell secretory protein (CCSP), and the mucus cell marker Muc5AC to characterize epithelial cell phenotypes. In agreement with previous studies [3], wild-type C57/Bl6 mouse tracheal submucosal glands were restricted to the first two intratracheal cartilaginous rings (Figures 1A, 1E and Supporting information, Supplementary Figures 1A and 1B). In contrast, the distance to which keratin 14-expressing SMGs extended was significantly increased throughout Myd88KO tracheas (Figures 1B, 1E and Supporting information, Supplementary Figures 1A and 1B). KO tracheas also contained significantly greater Clara cell abundance and CCSP staining intensity (Figures 1C, 1D and Supporting information, Supplementary Figures 1A–1C).

Further immunophenotyping of secretory cells was performed by immunostaining with CCSP plus the mucus antigen Muc5AC, and revealed that many Myd88KO tracheal CCSP-expressing cells were also Muc5AC-positive (yellow staining, arrowheads, Supporting information, Supplementary Figures 1D and 1E). These data were consistent with results that we obtained following histochemical staining using Alcian blue and PAS to identify acidic and basic mucins, respectively (Supporting information, Supplementary Figures 1F–1I). These differences were not observed beyond the primary tracheal bifurcation (data not shown). There was no difference between wild-type and Myd88KO basal or ciliated cell abundance as determined by keratin 14 and acetylated tubulin (ACT) staining (Figures 1C, 1D, 1F and data not shown).

On examination of newborn Myd88 heterozygous and KO tracheas, we found no differences in airway differentiation or SMG abundance (Supporting information, Supplementary Figures 2A–2F). Heterozygous and KO newborn airway SMGs were always restricted to the most proximal intratracheal cartilaginous rings (Supporting information, Supplementary Figure 2G) and there were no differences in mucus cell abundance or differentiation between newborn heterozygous and KO mice (Supporting information, Supplementary Figures 2E, 2F, and 2H).

**Figure 2:**
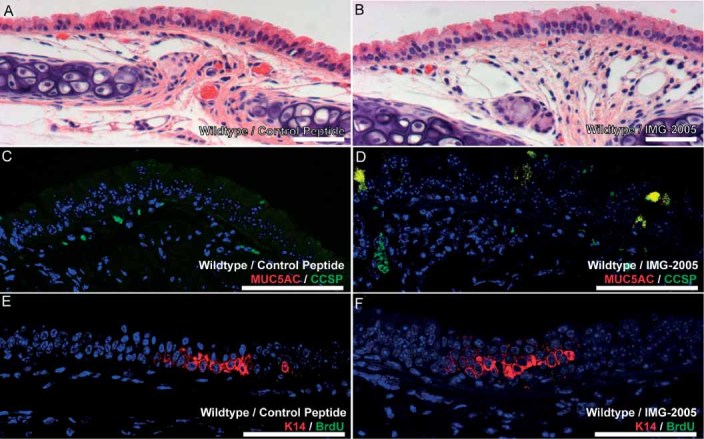
Pharmacological inhibition of Myd88 promotes mucus cell metaplasia. Representative images of control peptide (A, C, E) or IMG-2005-treated mice (B, D, F) stained with H&E (A, B), CCSP (green) plus Muc5AC (red; C, D), or K14 (red) plus BrdU (green; E, F) to assess overall epithelial appearance (A, B), mucus cell metaplasia (C, D), and epithelial cell proliferation (E, F). Five mice were treated in each group; scale bars are 100 µm (all panels)

Importantly, Myd88 deficiency may increase susceptibility to *in vivo* parasitic, bacterial, and viral infections, as well as alter normal inflammatory responses [29]. To prevent infection, all mice were housed in individually ventilated cages and subjected to routine pathogen screening. We also investigated whether Myd88KO tracheas exhibited altered infection or inflammation. Neither Gram staining nor immunohistochemical methods identified differences in bacterial colonization or local tissue inflammation in Myd88KO samples when compared with wild-type controls (data not shown) [30–32].

### Pharmacological inhibition of Myd88 is sufficientto promote mucous cell metaplasia

To assess whether pharmacological inhibition ofMyd88 was sufficient to promote our observed *in vivo* phenotypes, we treated adult wild-type mice systemically with the commercially available Myd88 inhibitory peptide IMG-2005 or a control peptide once daily for 10 days [33, 34]. Although H&E-stained sections of control and IMG-2005-treated wild-type mice appeared grossly similar (Figures 2A and 2B), dual immunofluorescent staining for CCSP plus Muc5AC revealed a significant increase in mucous cell abundance in IMG-2005-treated mice compared with controls (Figures 2C and 2D). There were no differences in K14-expressing cell abundance, nor were there differences in cell proliferation as assessed by BrdU incorporation among control peptide and IMG-2005-treated mice (Figures 2E and 2F). Finally, we observed no differences in submucosal gland abundance between control peptide and IMG-2005-treated mice. Overall, these results indicate that pharmacological inhibition of Myd88 is sufficient to promote mucous metaplasia in proximal murine airways.

### Wild-type and KO tracheal epithelial cells exhibit comparable *in vitro* differentiation

**Figure 3:**
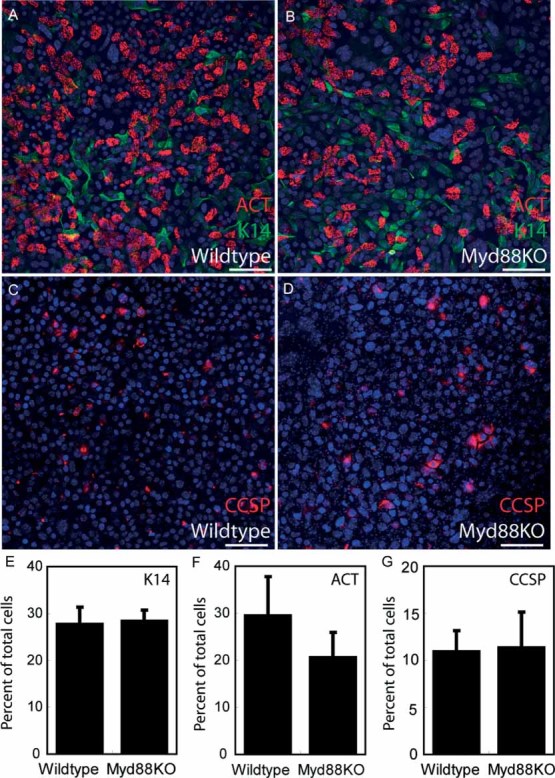
Wild-type and KO tracheal epithelial cells exhibit comparable *in vitro* differentiation. (A–D) Wild-type (A, C) and KO (B, D) tracheal epithelial cell cultures were grown at an air–liquid interface (ALI) that promotes differentiation closely resembling a normal *in vivo* epithelium. Differentiated ALI cultures were stained for K14 (green; A, B), acetylated tubulin (ACT; red, A, B), and CCSP (red; C, D) to assess differentiation. (E–G) K14-positive basal cells (E), ACT reactive ciliated cells (F), and CCSP-expressing Clara cells (G) all exhibited comparable abundance between wild-type and knockout samples as a function of total ALI surface area. Four ALI cultures per genotype were analysed; error bars represent standard error of the mean. All samples were also stained with DAPI nuclear dye (blue; A–D). Scale bars are 100 µm (A–D)

In order to determine whether Myd88 deficiency influenced intrinsic tracheal epithelial cell phenotype determination, we isolated and cultured adult wild-type and KO tracheal epithelial cells at an air–liquid interface (ALI). This technique causes rapid undifferentiated epithelial cell growth, followed by basal, ciliated, and secretory cell differentiation [24]. ALI cultures exhibit pseudostratification and closely resemble an *in vivo* tracheal epithelium [24]. There were no differences in proliferation between wild-type and Myd88KO cultures as determined by measuring epithelial density versus time (data not shown). ALI membrane immunostaining was used to assess wild-type and Myd88KO epithelial cell phenotypes. Both wild-type and KO tissues exhibited comparable pseudostratification and differentiation towards ciliated, basal, and secretory tracheal epithelial cell lineages (Figures 3A–3G). It should be noted, however, that it has previously been shown that murine ALI cultures undergo only limited secretory cell differentiation [24]. Despite this caveat, our results indicate that Myd88 deficiency does not alter *in vitro* epithelial cell proliferation or differentiation.

### Myd88 is required for normal tracheal epithelial resolution from injury

**Figure 4:**
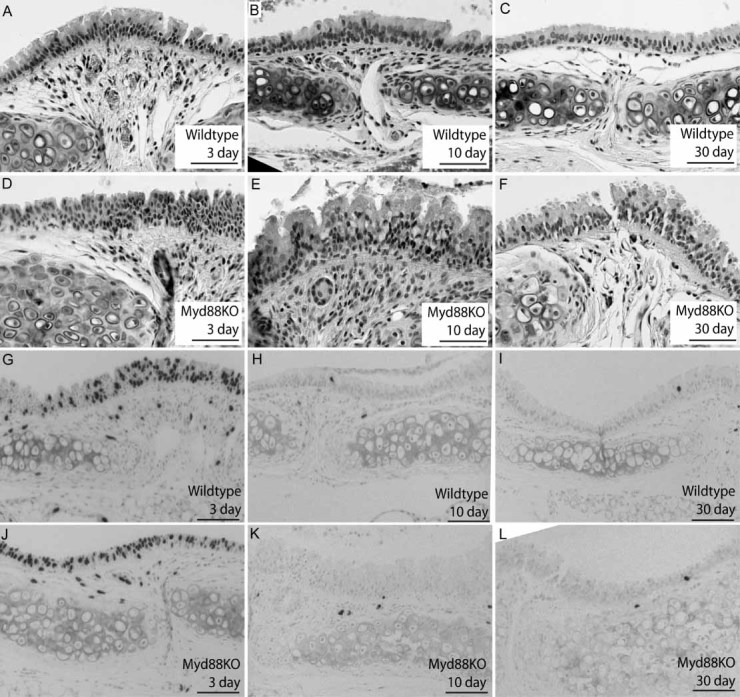
Myd88-deficient tracheas exhibit sustained hyperplasia but comparable proliferation after injury. (A–F) H&E staining of representative wild-type (A–C) and KO (D–F) tracheal sections from polidocanol-injured mice recovered for 3 (A, D), 10 (B, E), or 30 days (C, F). We observed both increased epithelial height and cellularity in Myd88KO tracheas relative to wild types at 10 and 30 days post-injury. (G–L) We also examined bromodeoxyuridine (BrdU) immunoreactivity (black; nuclear staining) to identify mitotic epithelial cells in wild-type (G–I) and KO airways (J–L) 3 (G, J), 10 (H, K), or 30 days after tracheal damage (I, L). There were no significant differences in epithelial proliferation between wild-type and KO airways at any time after epithelial injury. A minimum *n* = 3 mice were used for each recovery time point, and scale bars are 100 µm (all panels). Haematoxylin was used as a nuclear counterstain (G–L)

We next investigated whether Myd88 deficiency influenced resolution or repair following acute tracheal injury. To test this, adult mice were administered 2% polidocanol by oropharyngeal instillation to damage their epithelium and were allowed to recover for 3, 10, or 30 days. This method results in the rapid desquamation of tracheal epithelial cells within 24 h, followed by robust proliferation after 48–72 h and restoration of a phenotypically normal tracheal epithelium within 7–10 days [22]. Although H&E staining revealed that both wild-type and KO tracheas exhibited hyperplasia 3 days after injury, WT hyperplasia was largely resolved after 10 days and undetectable 30 days post-injury (Figures 4A–4C). In contrast, KO hyperplasia persisted throughout the entire recovery period (Figures 4D–4F).

These differences in epithelial hyperplasia were not due to altered cell proliferation or apoptosis. Control, 3-, 10-, and 30-day-recovered wild-type and Myd88KO mice were injected with 10 mg/kg BrdU 1 h prior to sacrifice and tissue sections stained using a BrdU-specific antibody (Figures 4G–4L). We observed robust epithelial BrdU staining 3 days after injury in both wild-type and KO tracheas (Figures 4G and 4J), with no evidence of continued proliferation at 10 and 30 days post-damage (Figures 4H, 4I, 4K, and 4L). Adjacent sections of WT and KO tracheas were stained with TUNEL to identify apoptotic cells at each of these time points but were equivalently low in WT and KO mice (Supporting information, Supplementary Figures 3A–3H).

**Figure 5:**
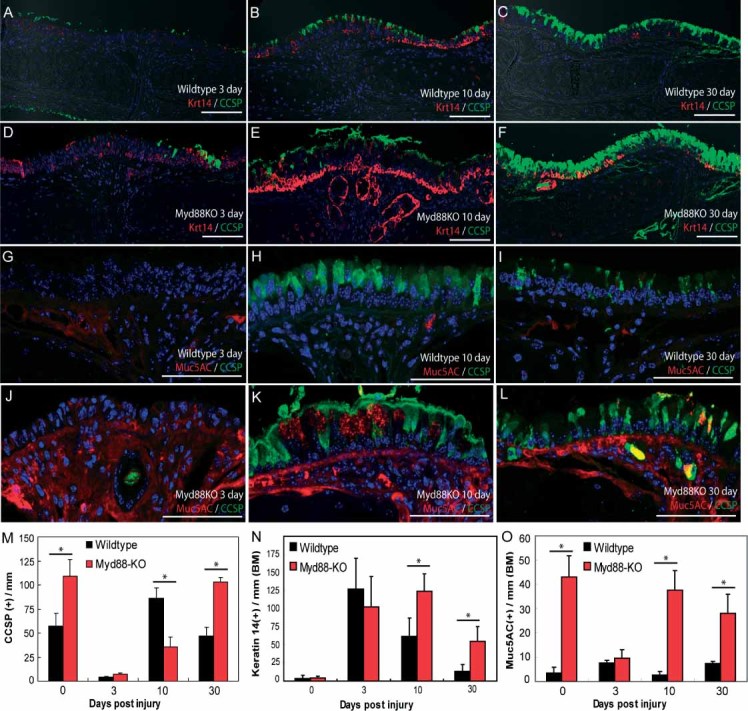
Myd88 expression is required for normal resolution of tracheal epithelial injury. (A–L) Representative confocal images of K14 (red; A–F) plus CCSP (green; A–F) or Muc5AC (red; G–L) plus CCSP (green; G–L) dual immunofluorescence staining on 2% polidocanol-injured wild-type (A–C, G–I) and Myd88KO (D–F, J–L) tracheas recovered for 3 (A, D, G, J), 10 (B, E, H, K), and 30 days (C, F, I, L). (M–O) Quantification of wild-type (black bars) and Myd88KO (red bars) tracheal epithelial cell phenotypes before and after polidocanol injury. We observed significant differences between WT and Myd88KO tracheal CCSP abundance 0, 10, and 30 days after injury (M); K14 abundance 10 and 30 days post-damage (N); and Muc5AC reactivity after 0, 10, and 30 days (O). Error bars (M–O) represent the standard error of the mean from a minimum *n* = 3 mice per experimental time point. Asterisks (M–O) denote significance at *p* < 0.05 as determined by ANOVA and post-test pairwise analysis. Scale bars (A–L) are 100 µm. BM denotes basement membrane

To determine whether KO-specific tracheal epithelial hyperplasia was due to abnormal metaplasia or ongoing epithelial-to-mesenchymal transition (EMT) processes, we examined mucus, Clara, basal, and smooth muscle actin (SMA)-expressing cell abundance 3, 10, and 30 days after injury (Figure 5 and Supporting information, Supplementary Figure 4). Consistent with previous results [22], wild-type tracheal epithelial CE cell abundance was reduced 3 days after injury, recovered within 10 days, and was associated with transient keratin 14-expressing basal cell hyperplasia (Figures 5A–5C). In contrast, polidocanol-damaged Myd88KO tracheas exhibited delayed CE cell restoration, persistent basal cell hyperplasia, and eventual CCSP overproduction at 3–30 days (Figures 5D–5F). Similarly, Myd88KO tracheas exhibited excessive Muc5AC production at later time points (Figures 5G–5L and 5O). CCSP, K14, and Muc5AC cell staining confirmed that KO tracheal epithelial cell phenotypes were significantly different from wild types(Figures 5M–5O). Assessment of cellular EMT phenotypes by SMA immunostaining at all time points revealed no significant differences between WT and Myd88KO tracheas (Supporting information, Supplementary Figure 4). In contrast, SMA staining was always restricted to the myoepithelium surrounding epithelial submucosal gland cells.

### Uninjured Myd88-deficient tracheas exhibita unique gene expression profile

**Figure 6:**
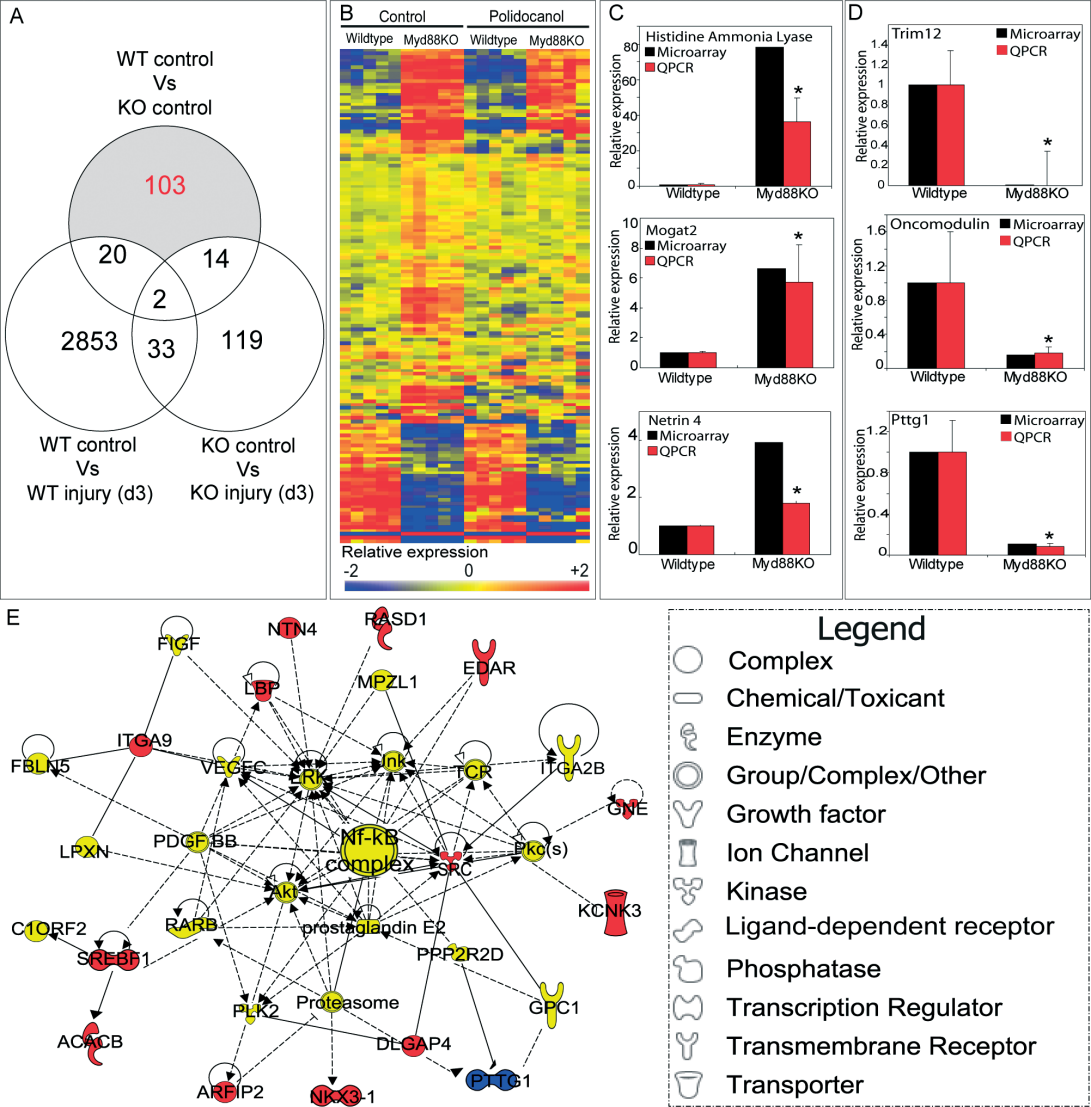
Uninjured Myd88-deficient tracheas exhibit a unique gene expression profile. (A) Venn diagram of array samples, comparison conditions, and transcript clustering. Numbers indicate differentially expressed probes that are uniquely expressed for each comparison condition. Only transcripts that were uniquely differentially expressed in uninjured Myd88KO tracheas relative to all other categories (uninjured wild-type, polidocanol-injured Myd88KO, and polidocanol-injured wild-type) were considered (103 transcripts in total; shaded region). (B) Heat maps representing relative expression of selected transcripts in uninjured WT versus Myd88KO samples. Blue and red colours depict low and high expression levels relative to a normalization of all array samples, respectively. (C, D) Quantitative RT-PCR analysis of relative mRNA expression for up-regulated (C) and down-regulated (D) genes identified by microarray in uninjured knockout and wild-type tracheal samples. Knockout tracheal RNA levels (red bars) were consistent with microarray results (black bars). (E) Top-ranked gene interaction network identified using Ingenuity Pathways Analysis (IPA) software derived from selected differentially expressed transcripts. Gene function is depicted by shape (see legend in figure); colours correspond to expression in Myd88KO samples relative to WT (see Supporting information, Supplementary Table 1). Solid and dashed lines denote previously identified direct and indirect gene interactions, respectively. Error bars (C, D) represent the standard error of the mean (*n* = 4 samples per genotype); asterisks denote significance at *p* < 0.05

To elucidate mechanisms that may explain this hypersecretory phenotype, we used wild-type and Myd88KO gene expression profiling. We compared ten WT and ten KO whole tracheal samples (five control and five 3-day post-injury) using Agilent gene expression microarrays (GEO series GSE17268). Genespring X7.3 software was used to screen 41,267 total transcripts, and samples exhibiting a minimum statistical significance of *p* < 0.01 were considered to be differentially expressed. Using these criteria, 2908 transcripts exhibited differential expression between wild-type uninjured controls and injury samples; 168 between KO uninjured controls and KO injury; and 139 between WT controls and KO controls (Figure 6A). Because we were interested in identifying mechanisms that might explain the Myd88KO tracheal epithelial phenotype present in uninjured mice, we excluded all transcripts that exhibited differential expression in injured WT and injured KO samples relative to controls. The remaining gene subset included 103 transcripts representing 53 significantly up-regulated genes plus 19 down-regulated genes (*p* < 0.01; Figure 6A, shaded area; Figure 6B; and Supporting information, Supplementary Table 1). The top ten most significantly up- and down-regulated genes from this list are presented in Table 1.

**Table 1:** Top ten up- and down-regulated genes in uninjured knockout samples relative to uninjured wild-type samples

Rank	Gene name	Abbreviation(s)	Gene identifier	Fold change
1	Histidine ammonia lyase	*Hal, Hsd*	NM_010401	78.24
2	Integrin alpha 9	*Itga9*	NM_133721	10.26
3	CUE domain containing 1	*Cuedc1*	NM_198013	9.499
4	Monoacylglycerol O-acyltransferase 2	*Mogat2, Mgat2*	NM_177448	6.623
5	Myosin, heavy polypeptide 7	*Myh7, Myhcb*	NM_080728	5.003
6	Glucocorticoid induced gene 1	*Gig1, Zfp704*	NM_133218	4.901
7	Oogenesin 1	*Oog*	NM_178657	4.766
8	Arylsulphatase K	*Arsk*	NM_029847	4.428
9	Neuronal pentraxin 2	*Nptx2, Narp*	NM_016789	4.19
10	Netrin 4	*Ntn4*	NM_021320	3.918
1	Tripartite motif protein 12	*Trim12*	NM_023835	− 136.61
2	Tripartite motif protein 16	*Trim16, Ebbp*	NM_053169	− 101.63
3	Pituitary tumour transforming growth factor 1	*Pttg1, Pttg*	NM_013917	− 9.346
4	Arylsulphatase K	*Arsk*	NM_029847	− 7.353
5	Endoplasmic oxidoreductase 1 beta	*Ero1lb*	NM_026184	− 6.711
6	Cell cycle related, elevated tumour protein	*Crept*	NM_027434	− 6.711
7	Oncomodulin	*Ocm*	NM_033039	− 6.329
8	FYVE/coiled-coil domain containing 1	*Fyco1, Mem2*	NM_148925	− 4.808
9	Tripartite motif protein 34	*Trim34*	NM_030684	− 4.566
10	Tyrosine sulphotransferase	*Sult1b1*	NM_019878	− 4.444

Fold change represents relative expression in uninjured KO control samples relative to WT control samples. Genes were selected based on a statistical significance of at least *p* < 0.01 (*n* = 5) and a lack of further differential expression in the context of epithelial injury (see Figure 5A).

We validated representative differentially expressed genes identified in this study using quantitative RT-PCR analysis of uninjured whole tracheal samples (Figures 6C and 6D). Uninjured Myd88KO samples expressed significantly increased histidine ammonia lyase (*Hal*), monoacylglycerol O-acyltransferase 2 (*Mogat2*), and netrin 4 (*Ntn4*) relative to uninjured wild-type samples (Figure 6C). Myd88KO samples also expressed significantly decreased tripartite motif 12 (*Trim12*), oncomodulin (*Ocm*), and pituitary tumour transforming gene 1 (*Pttg1*) expression versus uninjured wild-type controls (Figure 6D). All of these findings were in good agreement with the microarray results. Many of the genes that we identified (including all genes validated by RT-PCR) have previously been identified as regulators of epithelial metaplasia or differentiation.

To further characterize the Myd88KO gene expression profile, we used Ingenuity Pathways Analysis (IPA) software (Ingenuity Systems, Redwood City, CA, USA). IPA software collates findings presented in peer-reviewed publications into networks of physical, transcriptional, or enzymatic interactions to reveal functional and mechanistic pathway associations. The most significant of these was associated with NF-κB activation and included genes encoding the cell surface ectodysplasin A receptor (Edar) and lipopolysaccharide binding protein (Lbp). Interestingly, both of these genes have previously been functionally associated with epithelial mucus production and submucosal gland differentiation [8, 35, 36] (Figure 6E).

**Figure 7:**
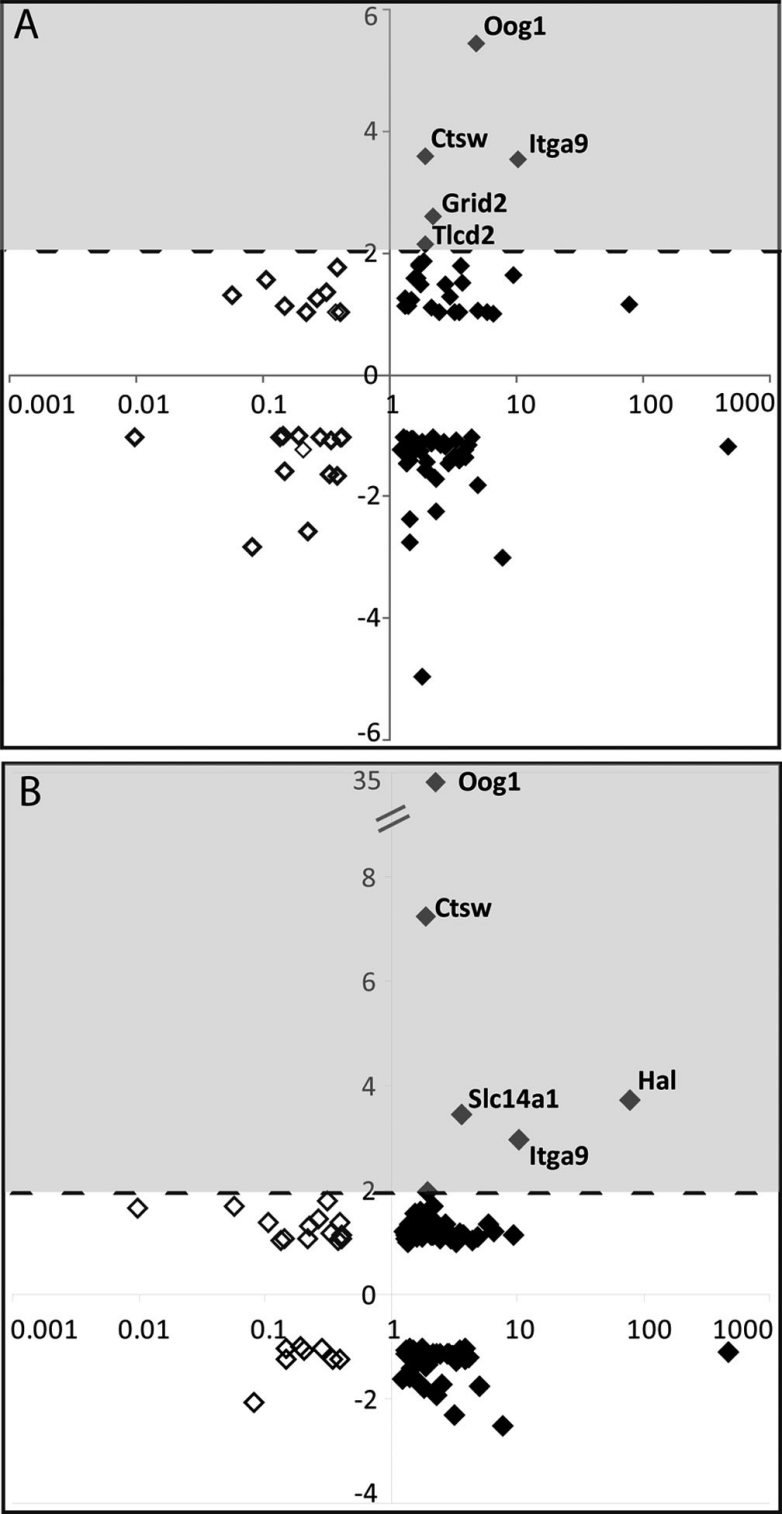
Genes associated with Myd88 deficiency are not enriched in tracheal epithelial stem and progenitor cells. Differentially expressed genes identified in our microarray studies (Figure 6A and Supporting information, Supplementary Table 1) were compared with publicly available tracheal epithelial stem and progenitor cell gene expression profiles (GSE15724). (A, B) Uninjured Myd88KO tracheal gene expression relative to wild-type samples is plotted logarithmically on the *X*-axis. Up- and down-regulated genes are depicted by filled and clear diamonds, respectively. Each gene was also plotted along the *Y*-axis according to its enrichment in defined tracheal stem (A) or progenitor (B) cells relative to a negatively staining cell population. We only considered genes exhibiting a minimum two-fold enrichment in either tracheal stem or progenitor cell populations (shaded areas, A and B). Using these criteria, only three out of 103 transcripts identified in our studies were additionally enriched in tracheal stem and progenitor cells

It has been suggested that endogenous tracheal epithelial stem or progenitor cells drive submucosal gland abundance [37]. We would therefore expect that some differentially expressed genes identified in the Myd88 KO tracheas might overlap with genes differentially expressed in epithelial progenitor cells. We performed a comparative microarray analysis between our dataset (Supporting information, Supplementary Table 1) and that of a recent study [27] (Figure 7). Each gene identified in our studies was plotted on the *X*-axis based on its expression in knockout tissue relative to uninjured wild-type samples. Along the *Y*-axis, we plotted expression of these same genes based on their expression in either tracheal lectin plus keratin 5-expressing (stem) or lectin-only (progenitor) cell populations (Figures 7A and 7B, respectively). To remain consistent with previous analyses [27], we only considered genes that exceeded a minimum two-fold, statistically significant enrichment (shaded boxes, Figures 7A and 7B). The results revealed that only 5 of 103 differentially expressed transcripts were significantly enriched in tracheal stem or progenitor cell populations, suggesting that the majority of Myd88-dependent transcriptional changes do not occur within tracheal stem or progenitor cells.

## Discussion

We have characterized adult tracheal epithelial metaplasia in the context of Myd88 deficiency. Myd88 deletion drove increased submucosal gland abundance, mucus cell metaplasia, elevated CCSP and Muc5AC expression, and Clara cell hyperplasia. These phenotypes were not associated with persistent tracheal infection or inflammation (as judged by Gram stain and inflammatory cell infiltration) and were also not present during embryogenesis. The observed adult tracheal mucous cell metaplasia phenotype was recapitulated following systemic administration of the commercially available Myd88 inhibitory peptideIMG-2005. This finding strongly implicates the Myd88/NF-κB pathway as a central mediator of airway homeostasis and mucus production. Myd88 was also required for normal resolution from epithelial injury. Interestingly, however, Myd88-deficient cells exhibited normal *in vitro* differentiation, and microarray studies found little overlap between previously published epithelial progenitor cell transcripts and those differentially expressed in the Myd88 KO trachea.

We observed increased submucosal gland formation, mucus cell abundance, and epithelial hyperplasia only within tracheas, despite global Myd88 deletion. This result was consistent with the observation that murine mucus hypersecretion and mucus cell transdifferentiation are limited to proximal airways, despite the use of injury models that target the whole lung [4]. Additionally, with no evidence of persistent infection or inflammation in the Myd88KO mice, we found that Myd88KO tracheas exhibited abnormal repair and increased secretory protein production following injury. Given that both CCSP and mucin proteins are known to modulate airway oxidative damage, our findings suggest that Myd88 may regulate tracheal stress responses and subsequent airway re-epithelialization after injury [1, 4].

Our data are consistent with previous reports linking Myd88, NF-κB activity, and initial lung epithelial differentiation [7, 11, 38, 39]. It is accepted that NF-κB signalling regulates distinct airway epithelial processes including differentiation, hyperplasia, and appendage formation. In our study, Ingenuity Pathways Analysis identified enrichment of NF-κB signalling pathway members in KO tracheal samples. This suggests that NF-κB may modulate adult tracheal epithelial metaplasia. Our results are also consistent with earlier studies suggesting that non-epithelial signalling regulates tracheal epithelial differentiation [40]. Specifically, we found that both KO and wild-type epithelial cells exhibited comparable intrinsic differentiation capacity when grown at an *in vitro* air–liquid interface. This result suggests that extrinsic, rather than epithelial cell autonomous or cell-intrinsic, factors influence adult tracheal secretory cell phenotypes.

Ingenuity Pathway Analysis performed as part of this study identified several gene-interaction networks that are differentially regulated in KO tracheas and may mechanistically explain our observed phenotypes. These networks included the genes *Edar* and *Lbp*, each of which are known regulators of lung epithelial cell phenotype. Ectodysplasin A (*Eda*) and *Edar*-deficient mouse strains (Tabby, downless, crinkled) lack submucosal gland development, despite normal basal keratinocyte progenitor cell abundance [8]. Separately, *Lbp* knockout mice fail to exhibit reduced airway hyperreactivity and mucus cell metaplasia after endotoxin exposure [36]. *Lbp* has also been shown to directly regulate airway hyperreactivity by facilitating lipopolysaccharide binding to myeloid cells [41].

Microarray analysis revealed differential expression of 103 transcripts in Myd88KO versus WT tracheas. Differentially expressed genes included both uncharacterized and previously identified regulators of epithelial cell phenotypes. We assessed the expression of six of these genes in adult tracheas using quantitative RT-PCR (QPCR). Three of these genes (*Mogat2, Hal*, and *Ntn4*) exhibited significant up-regulation. It has previously been shown that *Mogat2*-deficient mice exhibit numerous epithelial defects including overabundant intestinal epithelial mucus production [42]. Separately, *Hal* gene expression has been associated with altered skin epithelial cell differentiation [43]. Netrin 4 has also recently been shown to influence lung epithelial outgrowth formation in a genetically modified mouse model [44]. Down-regulated genes validated by QPCR included *Trim12, Ocm*, and *Pttg1*. Interestingly, these genes are also known regulators of epithelial homeostasis and differentiation [45–47]. Specifically, *Trim* family members are required for maintenance of skin epithelial cells in an undifferentiated state [32], whereas *Ocm* and *Pttg1* are both modulators of epithelial growth and regeneration [33, 34, 48].

This report is the first to identify a role for Myd88 in adult tracheal submucosal gland abundance. Overall, the results of this study, alongside those of previous publications, suggest a complex mechanism of signalling network interactions that regulates postnatal tracheal submucosal gland development, mucus cell homeostasis, and metaplasia.

## SUPPORTING INFORMATION ON THE INTERNET

The following supporting information may be found in the online version of this article.

Supplementary methods

**Figure S1.** Further characterization of Myd88-deficient tracheal cell phenotype.

**Figure S2.** Newborn KO tracheas exhibit normal differentiation.

**Figure S3.** Myd88-deficient tracheas do not exhibit altered epithelial apoptosis after epithelial injury.

**Figure S4.** Wild-type and Myd88KO tracheas exhibit comparable smooth muscle actin cell abundance.

**Table S1.** Differentially regulated genes in uninjured Myd88-deficient tracheas.
