# Supplementary figures and images for: Myd88 deficiency influences murine tracheal epithelial metaplasia and submucosal gland abundance
Source: J Pathol. 2011 May 10;224(2):190–202. doi: 10.1002/path.2876 (PMC3434371; doi:10.1002/path.2876)

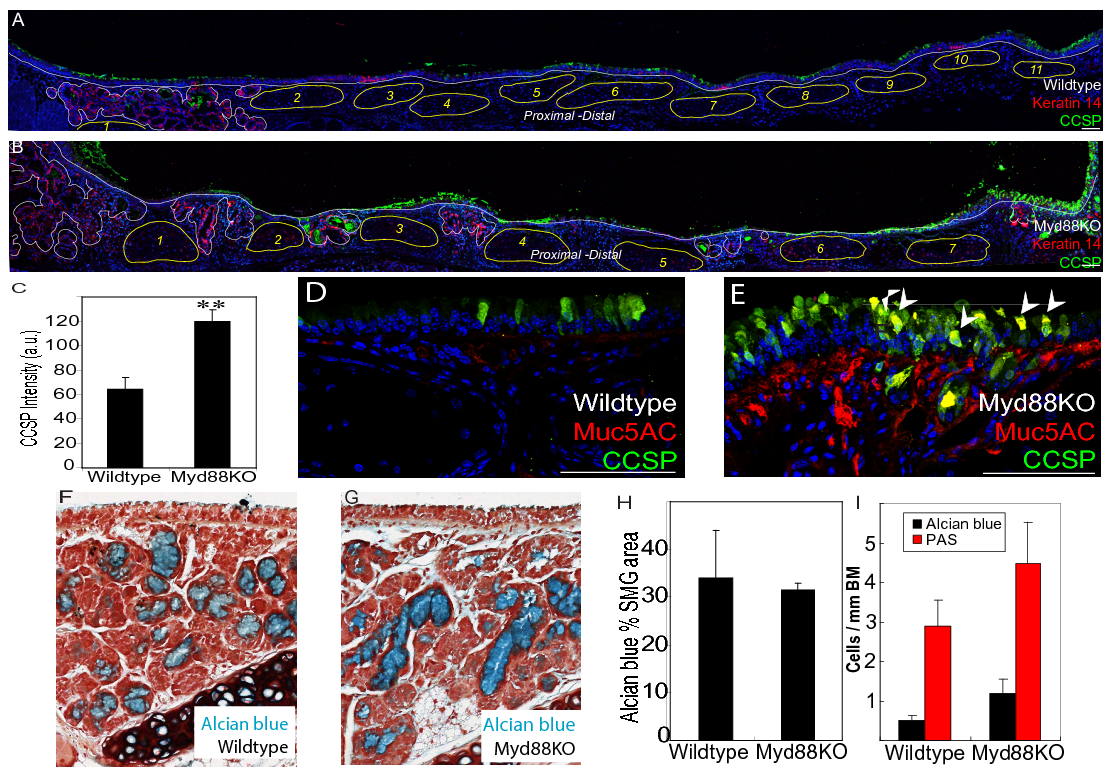

Supplement: Supplementary file 1 [file path0224-0190-SD1.tif]

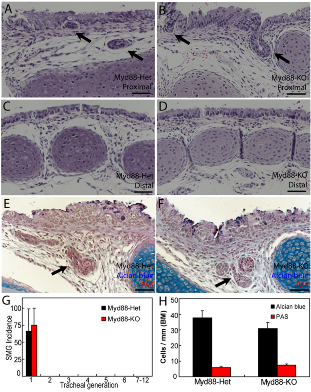

Supplement: Supplementary file 2 [file path0224-0190-SD2.tif]

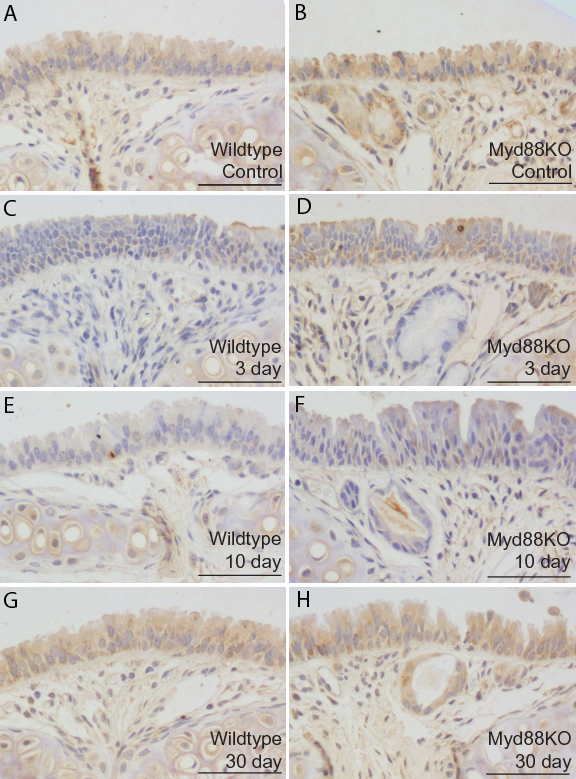

Supplement: Supplementary file 3 [file path0224-0190-SD3.tif]

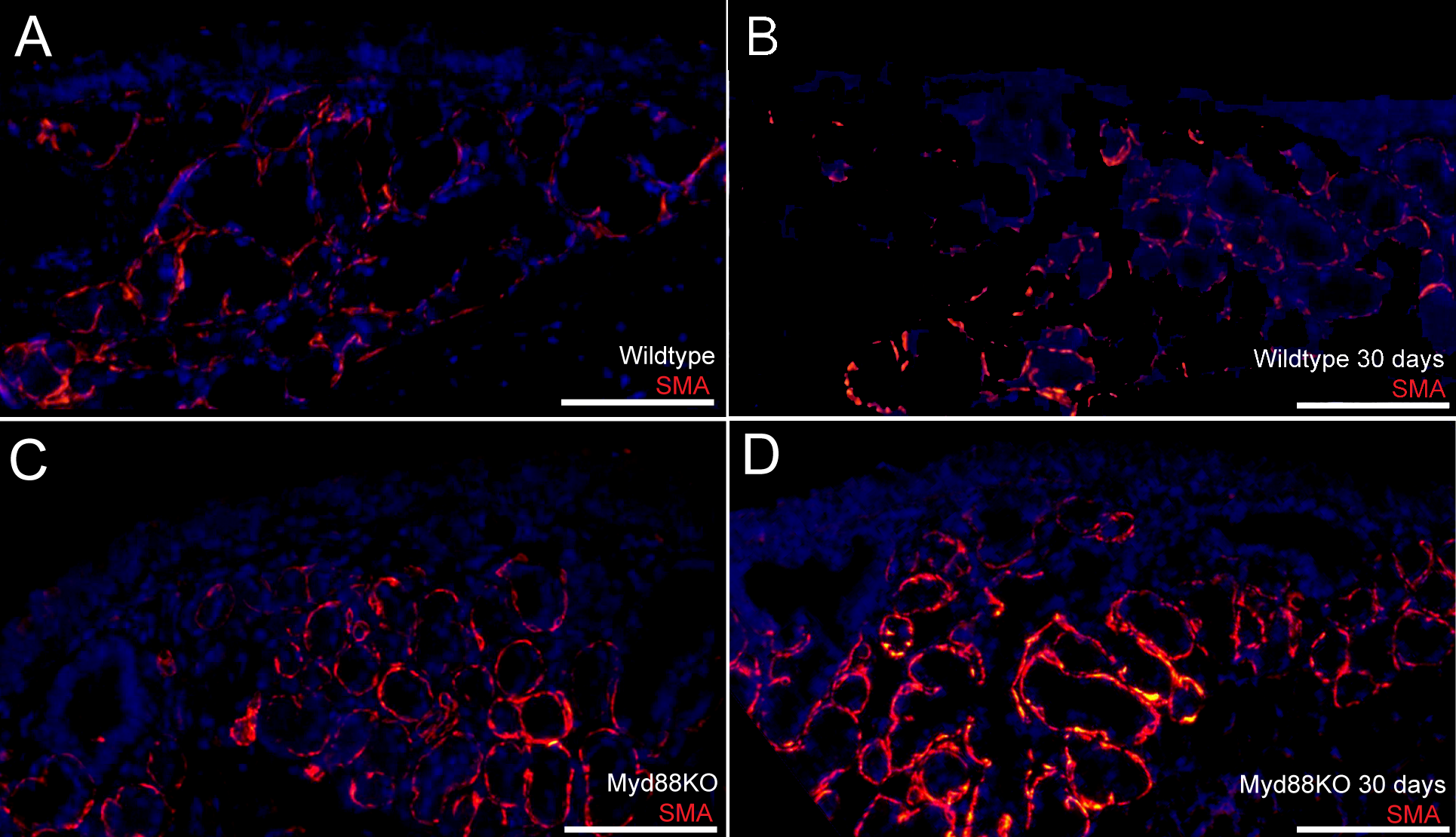

Supplement: Supplementary file 4 [file path0224-0190-SD4.tif]
