# Supplementary material for: Myd88 deficiency influences murine tracheal epithelial metaplasia and submucosal gland abundance
Source: J Pathol. 2011 May 10;224(2):190–202. doi: 10.1002/path.2876 (PMC3434371; doi:10.1002/path.2876)
Supplement: Supplementary file 5 [file path0224-0190-SD5.doc]

**Supporting information**

**Supplementary Table 1.** Differentially regulated genes in uninjured Myd88-deficient tracheas

| **Affymetrix ID** | **Fold Change** | **Gene Identifier** | **Gene Abbreviation** | **Gene Name** |
| --- | --- | --- | --- | --- |
| A_51_P172155 | 78.24 | NM_010401 | Hal | histidine ammonia lyase |
| A_51_P382970 | 10.26 | NM_133721 | Itga9 | integrin alpha 9 |
| A_51_P379409 | 9.50 | NM_198013 | Cuedc1 | CUE domain containing 1 |
| A_51_P411345 | 6.62 | NM_177448 | Mogat2 | monoacylglycerol O-acyltransferase 2 |
| A_52_P88033 | 5.00 | NM_080728 | Myh7 | myosin, heavy polypeptide 7, cardiac muscle, beta |
| A_52_P414138 | 4.90 | NM_133218 | Zfp704 | glucocorticoid induced gene 1 |
| A_51_P409250 | 4.77 | NM_178657 | Oog1 | oogenesin 1 |
| A_52_P475080 | 4.43 | NM_029847 | Arsk | arylsulfatase K |
| A_52_P187058 | 4.19 | NM_016789 | Nptx2 | neuronal pentraxin 2 |
| A_51_P497661 | 3.92 | NM_021320 | Ntn4 | netrin 4 |
| A_52_P642167 | 3.91 | NM_009230 | Soat1 | sterol O-acyltransferase 1 |
| A_52_P395228 | 3.78 | NM_008710 | Nnt | nicotinamide nucleotide transhydrogenase |
| A_51_P312336 | 3.69 | NM_028122 | Slc14a1 | solute carrier family 14 (urea transporter), member 1 |
| A_51_P512210 | 3.57 | NM_010856 | Myh6 | myosin, heavy polypeptide 6, cardiac muscle, alpha |
| A_52_P159050 | 3.52 | BC059776 | Cds2 | Cds2 protein |
| A_51_P469942 | 3.32 | NM_146386 | Myocd | myocardin isoform B |
| A_51_P227866 | 3.29 | BC054558 | Txndc13 | Unknown (protein for MGC:62508) |
| A_52_P616332 | 3.23 | NM_153389 | Atp10d | ATPase, Class V, type 10D |
| A_51_P129895 | 2.99 | NM_007744 | Comt | catechol-O-methyltransferase |
| A_51_P514405 | 2.91 | NM_019741 | Slc2a5 | solute carrier family 2 (facilitated glucose transporter), member 5 |
| A_51_P454008 | 2.76 | NM_008489 | Lbp | lipopolysaccharide-binding protein |
| A_51_P404193 | 2.70 | NM_022435 | Sp5 | trans-acting transcription factor 5 |
| A_52_P367034 | 2.47 | NM_030684 | Trim34 | tripartite motif protein 34 |
| A_52_P16419 | 2.31 | NM_010271 | Gpd1 | glycerol-3-phosphate dehydrogenase 1 (soluble) |
| A_51_P226453 | 2.23 | NM_025590 | Acot11 | thioesterase, adipose associated |
| A_52_P303491 | 2.18 | AK032669 | Grid2 |  |
| A_51_P520718 | 2.17 | NM_011196 | Ptger3 | prostaglandin E receptor 3, subtype EP3 |
| A_51_P220262 | 2.17 | XM_129042 | Rnf165 | similar to ring finger protein 165 |
| A_51_P486512 | 2.11 | NM_134093 | Letmd1 | cervical cancer receptor |
| A_52_P319180 | 1.96 | NM_009724 | Atp4b | ATPase, H+/K+ transporting, beta polypeptide, gastric specific |
| A_52_P264924 | 1.91 | NM_009985 | Ctsw | cathepsin W preproprotein |
| A_51_P239236 | 1.89 | BC022940 | Acacb | Acacb protein |
| A_51_P161812 | 1.84 | NM_010100 | Edar | ectodysplasin-A receptor |
| A_51_P228193 | 1.68 | NM_023429 | Ociad1 | OCIA domain containing 1 |
| A_51_P419117 | 1.60 | NM_134050 | Rab15 | RAB15, member RAS oncogene family |
| A_51_P360918 | 1.57 | NM_020578 | Ehd3 | EH-domain containing 3 |
| A_52_P188099 | 1.54 | AK033507 | Gne |  |
| A_51_P224530 | 1.49 | NM_020610 | Nrip3 | nuclear receptor interacting protein 3 |
| A_52_P14682 | 1.48 | AK143615 | Scfd2 |  |
| A_51_P352357 | 1.44 | NM_175349 | Ldhal6b | L-lactate dehydrogenase A-like |
| A_51_P109050 | 1.43 | NM_023824 | Paqr4 | progestin and adipoQ receptor family member IV |
| A_52_P545010 | 1.43 | NM_027216 | Slc39a11 | solute carrier family 39 (metal ion transporter), member 11 |
| A_51_P517012 | 1.39 | AK005069 | Tysnd1 |  |
| A_51_P140901 | 1.38 | NM_016881 | Pmm2 | phosphomannomutase 2 |
| A_52_P656565 | 1.38 | NM_145488 | Pex6 | peroxisomal biogenesis factor 6 |
| A_51_P448618 | 1.36 | AK165240 | Slc16a10 |  |
| A_52_P265877 | 1.34 | NM_019993 | Aldh9a1 | aldehyde dehydrogenase 9, subfamily A1 |
| A_51_P148814 | 1.33 | NM_008529 | Ly6e | lymphocyte antigen 6 complex, locus E |
| A_52_P635271 | 1.32 | NM_201646 | Btbd6 | BTB (POZ) domain containing 6 |
| A_51_P143468 | 1.32 | NM_178771 | Klhl26 | kelch-like 26 isoform 2 |
| A_51_P373428 | 1.31 | NM_172371 | Slc16a13 | solute carrier family 16 (monocarboxylic acid transporters), member 13 |
| A_51_P484764 | 1.29 | NM_025473 | 1810037C20Rik | family 3, member A protein |
| A_51_P326425 | 1.22 | NM_029802 | Arfip2 | ADP-ribosylation factor interacting protein 2 |
|  |  |  |  |  |
| A_51_P209873 | -2.42 | NM_024287 | Rab6 | RAB6A, member RAS oncogene family |
| A_51_P302056 | -2.43 | NM_019756 | Tubd1 | tubulin, delta 1 |
| A_51_P417321 | -2.45 | AK156842 | Zfp236 |  |
| A_52_P407145 | -2.57 | NM_025299 | Txnl4 | thioredoxin-like 4 isoform a |
| A_52_P641216 | -2.58 | NM_175154 | Galk2 | galactokinase 2 |
| A_52_P330488 | -2.65 | NM_011023 | Otx1 | orthodenticle 1 |
| A_52_P236088 | -2.98 | NM_025638 | Gdpd1 | glycerophosphodiester phosphodiesterase domain containing 1 |
| A_52_P120719 | -3.14 | NM_030554 | Rab27b | RAB27b, member RAS oncogene family |
| A_52_P232346 | -3.24 | TC1479950 |  |  |
| A_52_P48546 | -3.76 | NM_026259 | Rnf41 | ring finger protein 41 |
| A_51_P206551 | -4.44 | NM_019878 | Sult1b1 | dopa/tyrosine sulfotransferase |
| A_52_P278853 | -4.57 | NM_030684 | Trim34 | tripartite motif protein 34 |
| A_52_P180101 | -4.81 | NM_148925 | Fyco1 | FYVE and coiled-coil domain containing 1 |
| A_52_P634829 | -5.24 | NM_023835 | Trim12 | tripartite motif protein 12 |
| A_52_P79648 | -6.33 | NM_033039 | Ocm | oncomodulin |
| A_51_P229633 | -6.71 | NM_027434 | 2610304G08Rik | cell-cycle related and expression-elevated protein in tumor |
| A_52_P603836 | -6.71 | NM_026184 | Ero1lb | endoplasmic oxidoreductase 1 beta |
| A_51_P472531 | -7.35 | NM_029847 | Arsk | arylsulfatase K |
| A_51_P371190 | -9.35 | NM_013917 | Pttg1 | pituitary tumor-transforming 1 |
| A_52_P212650 | -101.63 | NM_053169 | Trim16 | tripartite motif protein 16 |
| A_52_P267391 | -136.61 | BC094899 | Trim12 | Trim12 protein |
