# Supplementary material for: Myd88 deficiency influences murine tracheal epithelial metaplasia and submucosal gland abundance
Source: J Pathol. 2011 May 10;224(2):190–202. doi: 10.1002/path.2876 (PMC3434371; doi:10.1002/path.2876)
Supplement: Supplementary file 6 [file path0224-0190-SD6.doc]

**Supporting information**

**Supplementary figure legends**

**Supplementary Figure 1**. Further characterization of Myd88-deficient tracheal cell phenotype. (A, B) Whole longitudinal wild-type (A) and Myd88KO (B) tracheal sections (n=5 each) were stained with keratin 14 (red) plus CCSP (green) and photographed using confocal microscopy to better visualise submucosal gland distribution and secretory cell phenotypes. (C) Quantitative confocal fluorescence analysis of average CCSP fluorescence intensity in wild-type and Myd88KO tracheas. Wild-type (D) and KO (E) tracheas were stained with Muc5AC to assess SMG abundance and epithelial cell phenotype. (F, G) Representative images of Alcian blue reactive wild-type (F) and knockout (G) tracheal submucosal glands. (H) Quantification of wild-type and knockout alcian blue abundance within individual tracheal submucosal glands. (I) Quantitative analysis of both Alcian blue and periodic acid Schiff reactive cell abundance in wild-type and Myd88KO tracheas. Asterisks (C) indicate significance at p<0.005; scale bars (A, B, D, E) are 100µm.

**Supplementary Figure 2.** Newborn KO tracheas exhibit normal differentiation. (A-D) Representative images of H+E stained proximal (A, B) and distal (C, D) tracheas from newborn Myd88 heterozygous (A, C) and KO littermates (B, D). (E, F) Alcian blue (blue/purple) plus PAS (red/brown) histochemical stains in representative heterozygous (E) and KO proximal tracheas (F). (G) Quantification of newborn heterozygous and KO tracheal SMG incidence relative to location. (H) Alcian blue and PAS reactive mucous cell abundance in newborn heterozygous and KO tracheas (black and red bars, respectively). Error bars (G, H) represent the standard error of the mean (n=6 mice / genotype). Arrows (A, B, E, F) denote newborn tracheal SMGs. Scale bars are 100µm (A-F).

**Supplementary Figure 3.** Myd88-deficient tracheas do not exhibit altered epithelial apoptosis after epithelial injury. (A-H) Representative images of TUNEL staining in wild-type (A, C, E, G) and Myd88KO (B, D, F, H) control (A, B) or polidocanol injured tracheas recovered for 3 (C, D), 10 (E, F) or 30 days (G, H). Only rare TUNEL-reactive cells were ever observed in all samples and timepoints (arrowheads, D, E). A total of 3 mice were examined at each injury timepoint; scale bars are 100µm and hematoxylin was used as a nuclear counterstain (all panels).

**Supplementary Figure 4.** Wild-type and Myd88KO tracheas exhibit comparable smooth muscle actin cell abundance. (A-D) Representative images of smooth muscle actin (SMA) staining (red) in wild-type (A, B) and Myd88-KO (C, D) tracheas in the absence of injury (A, C) or following polidocanol injury and 30 days recovery (B, D). SMA staining was only ever observed adjacent to tracheal submucosal gland epithelia. Scale bars are 100μm; DAPI was used as a nuclear counterstain.
