# Supplementary material for: Myd88 deficiency influences murine tracheal epithelial metaplasia and submucosal gland abundance
Source: J Pathol. 2011 May 10;224(2):190–202. doi: 10.1002/path.2876 (PMC3434371; doi:10.1002/path.2876)
Supplement: Supplementary file 7 [file path0224-0190-SD7.doc]

**Supporting information**

**Supplementary methods**

*Quantitative confocal fluorescence microscopy*

We analysed the relative intensity of CCSP expression within individual tracheal epithelial cells using established quantitative confocal fluorescence techniques. Briefly, the average fluorescence pixel intensity of each cell was obtained by tracing the total cell perimeter and performing an area intensity quantification using Volocity image analysis software. At least 30 individual cells were analysed for each sample in order to obtain an average mean intensity. All images were obtained on the same day using identical confocal settings and 8-bit image acquisition. Data is expressed in arbitrary fluorescence intensity units based on 8-bit (0-256) image intensities.

*Newborn Myd88KO tracheal samples*

Myd88KO and heterozygous littermates were used to study Myd88 deficiency in newborn mice. These were generated by crossing Myd88KO females with a Myd88 heterozygous male. All newborn tissue samples were collected within 24 hours of birth and genotyped after sample collection. Three separate newborn litters were analysed during the course of this study.

*Quantitative RT-PCR*

Total tracheal RNA samples from 4 uninjured wildtype and 4 uninjured Myd88KO mice were used for quantitative RT-PCR (QRT-PCR) validation of microarray results. All QRT-PCR analyses were performed using an Applied Biosystems 7900HT real time PCR machine, inventoried Taqman probes and 2x Master Mix (Applied Biosystems, UK), and standard Taqman reaction conditions (60°C annealing and extension, 95°C denaturation). All samples were run in duplicate for each probe and normalised against 18S, GAPDH, and beta actin internal loading controls to determine individual ∆Ct values. Duplicate samples exhibiting significantly different Ct values (>0.3 difference) were reanalysed in separate experiments. Data represents the mean relative expression +/- SEM for all Myd88KO samples relative to wildtype controls. Inventoried Taqman probes were as follows:

Name Probe ID

| 18S | 4352930E |
| --- | --- |
| GAPDH | Mm99999915_g1 |
| Beta actin | Mm00607939_s1 |
| Hal | Mm00456709_m1 |
| Mogat2 | Mm00624192_m1 |
| Trim12 | Mm00844231_s1 |
| Oncomodulin | Mm00712881_m1 |
| Pttg1 | Mm00479224_m1 |
| Netrin 4 | Mm00480462_m1 |
